# Domain-Specific Self-Efficacy Scales for Elementary and Middle School Students

**DOI:** 10.11621/pir.2024.0103

**Published:** 2024-03-15

**Authors:** Diana R. Akhmedjanova

**Affiliations:** *National Research University Higher School of Economics, Moscow, Russia*

**Keywords:** self-efficacy, reliability, validity, academic achievement, metacognition, self-regulated learning

## Abstract

**Background.:**

Self-efficacy refers to students’ perceived confidence in their ability to tackle learning tasks. Research shows that self-efficacy serves as an important predictor of academic achievement and relates to students’ academic success, self-regulated learning, and motivation. It is therefore important to understand how self-efficacy develops and manifests itself in Russian schoolchildren and relates to their academic achievement.

**Objective.:**

To establish evidence of the validity and reliability of domain-specific self-efficacy scales developed for elementary and middle school students.

**Design.:**

Messick’s unified framework was used to establish validity. The surveys were administered to elementary and middle school students in two regions of Russia.

**Results.:**

The pilot testing of the self-efficacy scales for elementary school, using exploratory (*n* = 972) and confirmatory (*n* = 972) factor analyses, resulted in a four-factor model, which was later confirmed with a different sample of elementary students (*n* = 1,392) with good reliability estimates (α = 0.75–0.82). The pilot testing of self-efficacy scales for middle school, using exploratory (*n* = 583) and confirmatory (*n* = 584) factor analyses, resulted in a three-factor model, showing excellent reliability estimates (α = 0.88–0.93).

**Conclusion.:**

The evidence of construct validity suggests that the domain-specific self-efficacy scales for elementary and middle school students can be recommended for use by researchers and practitioners. The article presents ideas for additional validation studies and future research using domain-specific self-efficacy scales.

## Introduction

Self-efficacy refers to students’ perceived confidence in their ability to successfully tackle a task (Anderman & Wolters, 2008; Bandura, 1994, 2006). It relates to students’ engagement with tasks and the types of strategies they use (Bandura, 1994; Pajares, 2002), as well as to their learning, motivation, achievement, and self-regulated learning (Bernacki et al., 2015; Cespedes et al., 2021; DiBenedetto & Schunk, 2022; Peura et al., 2019; Schunk & DiBenedetto, 2016; Talsma et al., 2018; Wood et al., 2022). Students with high self-efficacy tend to study hard, have high motivation and academic achievement, seek new opportunities to learn, regulate their own learning, interpret their academic failures due to a lack of sufficient effort, and perceive learning difficulties as challenges to overcome (Bandura, 1994, 2006; DiBenedetto & Schunk, 2022; Schunk & DiBenedetto, 2016). In contrast, students with low self-efficacy perceive their academic struggles as the result of low cognitive ability, avoid challenging tasks, and have low confidence in their capabilities to study well (Bandura, 1994, 2006).

Research has shown that children’s self-efficacy changes with age. That is, younger children tend to overestimate their capabilities and show higher self-efficacy; however, with age and cognitive development, children’s assessment of their ability to perform tasks improves. There is a general trend that as students get older and transition through school, their self-efficacy decreases (Schunk & DiBenedetto, 2016) and becomes more stable (Talsma et al., 2018). Self-efficacy is dynamic, and it changes depending on tasks, experiences of mastery, and successes or failures (DiBenedetto & Schunk, 2022). High self-efficacy tends to be a strong predictor of students’ achievement and success (Bernacki et al., 2015; Schunk & DiBenedetto, 2016; Talsma et al., 2018).

### Theoretical Framework

**Figure 1.**
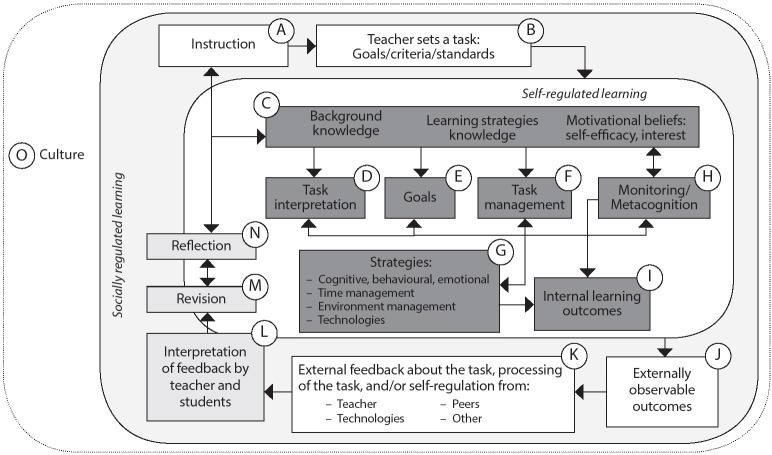
Model of self- and socially regulated learning

Historically, self-efficacy has been examined through the lens of social cognitive theory (Bandura, 1996) and described as a mutual interaction of personal, behavioral, and environmental factors (DiBenedetto & Schunk, 2022). In this study, we continue this tradition and situate self-efficacy within the *Model of Self- and Socially Regulated Learning* (Akhmedjanova, 2024; Figure 1), by recognizing the importance of personal, behavioral, and contextual factors. The model in Figure 1 is divided into three main sections: self-regulated learning (C–I, L–N), socially regulated learning (A–B, J–L), and culture (O). Instructional techniques (A–B) and formative assessment procedures (J–L) are examples of socially regulated learning (SoRL). Self-regulated learning (SRL) focuses on students’ background knowledge and motivational beliefs, including self-efficacy, which lead to their decision on which strategies to use to complete tasks (C–I, M–N). Finally, culture (O) places both SRL and SoRL in a sociocultural setting.

The model includes multiple processes that are activated when students work on their tasks. As part of instruction (A), a teacher sets a learning task (B), which activates students’ prior knowledge, knowledge of learning strategies, and motivational beliefs (C). For instance, if the task entails reading a chapter on quantum mechanics, students activate their prior knowledge of quantum mechanics, their interest in reading about this topic, the value (high vs. low) they place on it, and their level of self-efficacy in understanding this text. Students interpret tasks (D) in idiosyncratic ways, and their interpretations influence their personal goals and task management (F), as well as their self-efficacy and motivation (C). Based on their task interpretation, students set mastery or performance goals (E). A mastery goal for reading about quantum mechanics could be to apply new knowledge while conducting an experiment in the physics lab, which would suggest some positive prior experiences with the topic and high self-efficacy. Alternatively, a performance goal could be to pass the final physics test. Depending on their goals, students manage the completion of the task (F) by applying different strategies (G). For example, while reading about quantum mechanics, students can select a quiet place and specific time slots in which to read. They can choose to watch YouTube videos or use other online resources to understand complex parts of the text. While doing the task, students tend to monitor their progress on the task (H) and adjust the strategies they are using. That is, while reading the chapter on quantum mechanics, students might discover that they still do not understand some parts and can ask their teacher for clarification. As a result of the processes outlined in C–G, students develop internal learning outcomes (I) or, for instance, a better understanding of quantum mechanics.

Later, the internal outcomes are manifested in externally observable outcomes (J), such as students’ performance on tests or in lab experiments, which are assessed by teachers, peers, or technology (K). Feedback (K) provided by other sources identifies strengths and areas in need of improvement on the task, which contributes not only to the learning outcomes but also to students’ self-efficacy. Talsma and colleagues (2018) showed a reciprocal relationship between students’ performance and self-efficacy in their meta-analysis of 11 studies. That is, prior performance on a task relates to students’ self-efficacy, just as students’ self-efficacy relates to their future performance on similar tasks. Depending on students’ interpretations of the feedback (L), their levels of self-efficacy can become higher or lower in comparison with their self-efficacy at the task interpretation stage (D). Students can revise their task (M) after receiving feedback and overall reflection (N) on the process of learning, which can also contribute to changes in their self-efficacy (C), as evidenced in a study of 9th-grade students studying algebra (Bernacki et al., 2015). All the processes outlined in Figure 1 are situated within a complex context that brings together students and teachers with various cultural backgrounds^1^.

Self-efficacy plays an important role in the processes outlined in Figure 1, because if students feel self-efficacious, they are more motivated to do the task. Also, if they run into difficulties, these students are more likely to persist and try new strategies to complete the task and try similar ones in the future. As a result, it is important for teachers to know about students’ levels of self-efficacy in order to adjust instructional practices. To do so, both practitioners and researchers need psychometrically sound instruments to measure self-efficacy.

Numerous self-efficacy scales exist, such as the *Children’s Self-Efficacy Scale* (Bandura, 2006), the *Children’s Perceived Self-Efficacy Scale* (Jinks & Morgan, 1999), and the *Motivated Strategies for Learning Questionnaire* (MSLQ; Pintrich et al., 1993), which includes self-efficacy as part of the inventory. Hence, domain-specific self-efficacy scales have been extensively examined and have shown moderate to high relations of self-efficacy to achievement in those domains and to other psychological constructs such as school engagement or well-being. For instance, researchers measured self-efficacy for narrative writing (Grenner et al., 2021), self-efficacy for programming and its relationship to computational thinking (Wei et al., 2021), math self-efficacy mediated the path between teacher autonomy support and student engagement (Li et al., 2020), and general self-efficacy mediated the relationship between academic self-concept and subjective well-being in adolescents in Chile (Cespedes et al., 2021).

Self-efficacy is well-researched in Russia in the field of psychology among university students (Berman, 2020; Kotova et al., 2021). There are also a few studies examining the self-efficacy of Russian adolescents (Gordeeva & Shepeleva, 2006; Gorlova, 2020; Shepeleva, 2008). For example, Shepeleva (2008) found that 10^th^- and 11^th^-grade students (*n =* 156) with higher academic self-efficacy were more likely to use such coping strategies as active engagement. In addition, adolescents’ self-efficacy was related to their academic achievement. A more recent study of more than 15,000 Russian adolescents from PISA-2018 found that general self-efficacy mediated the relationship between reading and subjective well-being (Gorlova, 2020). The studies cited above focused on the academic and general self-efficacy of adolescent schoolchildren. However, Bandura (1994, 2006) stated that self-efficacy is dependent on the domain, and the level of specificity contributes to how students assess their confidence in performing certain tasks. In support of this position, Talsma et al. (2018) found that the effect sizes for relations between specific self-efficacy scales and academic performance are larger than for general self-efficacy scales. Therefore, in this study, we are proposing domain-specific self-efficacy scales for elementary and middle school students.

### Current Study

The primary goal of this study is to examine the psychometric properties of the self-efficacy scales developed for elementary and middle school students (American Educational Research Association [AERA] et al., 2014). The unified validity framework was used to establish the evidence of the construct validity of self-efficacy scales (Kane, 2006; Messick, 1995). The standards for educational and psychological testing outline five sources of validity evidence such as content, response processes, internal structure, relations with other variables, and consequences of testing, including the reliability of the scale (AERA et al., 2014; Messick, 1995).

To establish evidence of validity and reliability, we posed the following research questions:

1. What is the evidence of validity based on the content of the self-efficacy scales?
2. What is the evidence of validity based on the internal structure of the self-efficacy scales?
3. What is the evidence of validity based on the relations of the self-efficacy scales with other variables?
4. What is the evidence of reliability of the self-efficacy scales?

This paper attempts to provide evidence of construct validity by identifying: (1) content representation by describing the development of the scales; (2) evidence of internal structure by examining factor structure; (3) relations with other variables by examining convergent and discriminant evidence; and (4) reliability by examining the internal consistency of each scale. These sources of validity evidence are described in the following sections.

### Evidence Based on Content

Johnson and Morgan (2016) suggest developing new instruments in three successive phases: (1) operationalization of the construct; (2) pilot testing and scale development; and (3) survey model confirmation. Each phase is described in this paper.

#### Operationalization of Self-Efficacy

A group of experts in human development, self-regulated learning, and psychometrics examined available children’s self-efficacy scales (Bandura, 2006; Jinks & Morgan, 1999) to identify subscales and possible questionnaire items. However, none of the published instruments fit the Russian context for elementary school students. Therefore, we used Bandura’s guidelines (2006) to develop domain-specific self-efficacy scales. Bandura (2006) maintained that researchers should target “activity domains and assess the multifaceted ways in which efficacy beliefs operate within the selected activity domain” (p. 310). Given the evidence that specific self-efficacy scales relate better to academic performance (Talsma et al., 2018), we treated self-efficacy as domain-specific and developed separate self-efficacy scales by school subjects.

Neuropsychological evidence suggests that abstract thinking skills are still developing in elementary school children (Uytun, 2018). Therefore, we chose to phrase self-efficacy items in terms of whether students can or cannot do certain tasks within a domain. We consulted the federal state educational standards to identify core competencies that students should develop within each domain by the end of elementary school to align self-efficacy items with the competencies outlined in the standards. As a result, six self-efficacy scales were developed: math (4 items), writing (4), grammar (4), speaking (4), reading (4), and natural studies (5). A similar procedure was applied to the development of scales for foreign language (5 items), biology (4), and physics (5) for middle school students. The response scale for both elementary and middle school scales ranged from 1 (I cannot do it at all) to 4 (I can do it well) to facilitate a better understanding by students, even though the unipolar scales, ranging from 0 to 100, show better psychometric properties (Bandura, 2006; Talsma et al., 2018).

Before pilot testing, the initial cognitive laboratory was conducted with two fourth-grade students resembling the demographic characteristics of the target population, to check for the readability and students’ understanding of items on self-efficacy scales. Feedback from these students allowed us to revise some of the items to make them more age-appropriate and clear. Next, we carried out the pilot testing and survey model confirmation studies. The results of the pilot testing and survey model confirmation of self-efficacy scales for elementary school students are reported in the results section for Study 1.

The self-efficacy scales for middle school students (foreign language, biology, and physics) were pilot tested with another population in a different region of Russia. The results are reported in the results section for Study 2.

## Methods

This study is part of a longitudinal project, using a mixed-methods design to examine factors related to the academic failure of schoolchildren in Russia (https://ioe.hse.ru/failure-factors/). For the purposes of the present study, we used student data from the first wave collected in the fall of 2022 from fourth-grade students. Additionally, data collected for the research project implemented as part of the Basic Research Program at the National Research University Higher School of Economics (HSE University) were also used. Data were collected from students from public schools from the fourth through ninth grades in spring 2023.

### Participants

Three separate samples were used to conduct various analyses, depending on the instruments used in elementary and middle schools. To examine the instruments for elementary school, the sample from the longitudinal study included 1,944 fourth graders (49.74% girls) from a metropolitan city (*n* = 1,242), small towns (*n* = 554), and rural areas (*n* = 148). Another sample of 1,392 responses from elementary school students (50.14% girls), collected in spring 2023, was used for the confirmatory analyses. The analyses were performed on the data from students in the fourth (*n* = 406), fifth (*n* = 482), and sixth (*n* = 504) grades. To examine the instruments for middle school, the sample included 1,167 students (55.3% girls, *n* = 645) from seventh (*n* = 345), eighth (*n* = 514), and ninth (*n* = 308) grades.

### Instruments

In addition to the self-efficacy surveys evaluated in this study, the *Self-Regulated Learning [SRL] Strategies Survey for Elementary School Students* (Akhmedjanova & Lizunova, in press) and the metacognition scale (Lui et al., 2018) were used to check for relationships with other variables.

The SRL strategies survey is a 12-item scale focusing on strategies for managing environment, time, and learning, using a Likert-type scale (4 — almost always, 1 — almost never). Example item: *“I plan when I am going to do my homework.”* The internal consistency of the whole SRL scale is good, α = .83; ω_h_ = .71; ω_t_ = .85.

The metacognition scale is an adaptation of the SRL survey for the Diagnostic Assessment and Achievement of College Skills (DAACS, Lui et al., 2018). The survey includes the subscales of planning (5 items)^2^, monitoring (6 items)^3^, and reflection (3 items)^4^, using a Likert-type scale (4 — almost always, 1 — almost never). The confirmatory factor analysis (CFA) on Russian adolescents (*n =* 1167) confirmed the three-factor structure and indicated an excellent model fit, χ^2^ (74) = 447.01, *p* < .000, CFI = .99, TLI = .99, RMSEA = .07, SRMR = .04. The reliability estimates were good, α = .92; ω_h_ = .79; ω_t_ = .93.

### Procedure

After receiving approval from the HSE University’s Ethics Committee (#19), the data collection took place online in public schools in two regions of central Russia. Parents were informed about the purpose of the study and signed online consent forms, and children provided their assent to participate in this study.

### Data Analyses

The data analysis was conducted in R Studio. The missing data analyses were done using the *mice* package (van Buuren et al., 2022). The *psychometric* package (Fletcher, 2022) was used for the exploratory factor analysis (EFA), *lavaan* (Rosseel et al., 2023) for the CFA, and the *psych* package (Revelle, 2022) to run Pearson *r* correlation analyses and identify Cronbach’s alpha and McDonald’s omega reliability estimates.

### Missing Data

Missing data analyses were conducted for the fourth graders in the longitudinal study, revealing 0% missing data for students’ gender and location to 14% for self-efficacy in natural studies. The Pearson’s chi-squared test generated large *p*-values, which suggested that there was no association between missingness on the items for the self-regulation survey, self-efficacy for math, writing, grammar, reading, speaking, and natural studies, and the student’s gender. Additionally, the results indicated that the missingness mechanism was not systematic, and missing values were possibly missing completely at random (MCAR). Therefore, it was decided to use listwise deletion, which resulted in deleting 717 cases with missing values and reducing the sample size to 1,944 observations, which sufficed for further analyses.

A similar analysis was not performed on the sample of elementary school students in the second dataset (Study 2) because there was no missing data. However, the missing data analyses were conducted on the sample of 1,469 responses from students in grades 7 through 9. The analyses revealed various degrees of missing data depending on the variable, ranging from 0% for students’ gender and grade to 20% for the variable of self-efficacy for a foreign language. The Pearson’s chi-squared test generated large *p*-values, which suggested that there was no association between missingness on the items of the metacognition survey, self-efficacy for foreign language, biology, and physics, and the student’s gender and grade. Additionally, the results indicated that the missingness mechanism was not systematic, and missing values may have been missing completely at random. Therefore, it was decided to use listwise deletion, which resulted in deleting 302 cases with missing values and reducing the sample size to 1,167 observations that were used for analyses.

Since separate self-efficacy scales were developed for various school levels, the results are reported for elementary school in Study 1 and for middle school in Study 2.

## Results

### Study 1: Self-Efficacy Scales for Elementary School

The pilot testing phase was conducted on the data of the fourth-grade students. To establish validity evidence based on the internal structure, we conducted exploratory and confirmatory analyses.

#### Exploratory Factor Analysis

Self-efficacy scales by domain were developed for the purposes of the longitudinal project; therefore, both EFA and CFA were used to identify the factor structure. The sample from the longitudinal study (*n* = 1,944) was randomly split into two equal parts, which were used for EFA (*n* = 972) and CFA (*n* = 972). The EFA was conducted on the original six self-efficacy scales for math, writing, grammar, reading, speaking, and natural studies. The CFA allowed for verification of the factor structure proposed by the EFA.

Before conducting the EFA, the correlations and assumptions of factorability and sphericity were checked. The inter-item correlations indicated small to medium positive correlations among items (.14–.59). As expected, items within the same domains were more highly correlated with each other than with items from other domains. The Kaiser Meyer Olkin (KMO) factor adequacy overall estimate was .95, and the estimates for each item ranged from .91 to .97. The estimates of the Bartlett test of sphericity also suggested that a factor analysis was appropriate for this dataset, χ^2^ (300) = 3,560.48, *p* < .001.

The factor structure based on eigenvalues suggested a five-factor model; scree plots of the parallel analysis suggested a seven-factor model. Since the scale development included six distinct domains, the six-factor model was also checked. All models indicated a good model fit (*Table 1*); however, the factors for grammar and speaking did not work as expected. For example, in a five-factor model, the items for reading and speaking were loaded on one factor. Similarly, only two grammar items had loadings above 0.30 on a separate factor. As a result, it was decided to run a four-factor model. The results indicated a good model fit; therefore, it was decided to leave out the scales of grammar, speaking, and item 5 from the scale of self-efficacy for natural studies due to its low factor loading estimate.

**Table 1:** EFA Model Fit Indices (n = 972)

Model	χ^2^	*p*	TLI	RMSEA	RMSR
7-factor model	313.12	<.0001	.96	.03	.02
6-factor model	413.7	< .0001	.95	.04	.02
5-factor model	520.84	<.0001	.94	.04	.02
4-factor model	689.2	<.0001	.92	.05	.03

*Note. TLI — Tucker Lewis Index, RMSEA — Root Mean Square Error of Approximation, RMSR — Root Mean Square Residual*

#### Confirmatory Factor Analysis

The CFA analysis was conducted on the second half of the sample (*n* = 972) to examine the four-factor structure. The diagonally weighted least squares (DWLS) estimator was used to estimate the model parameters due to the ordinal nature of the self-efficacy scales. The CFA indicated an excellent model fit, χ^2^(98) = 292.02, *p* < .000, CFI = .99, TLI = .99, RMSEA = .04, SRMR = .04, with all items having medium to large factor loadings (.67–.89). The χ^2^/df coefficient resulted in an estimate of 2.97. x reports on the item-level statistics and Appendix A includes the self-efficacy items for elementary school. The survey confirmation study was conducted on a sample of the fourth, fifth, and sixth graders in spring 2023, using the scales of self-efficacy in math, writing, and reading. CFA revealed an excellent model fit, χ^2^ (51) = 264.09, *p* < .000, CFI = .99, TLI = .99, RMSEA = .05, SRMR = .04, with all items showing medium to large factor loadings. The χ^2^/df coefficient resulted in 5.17.

**Table 2:** Reliability Indices and Item Levels Estimates after CFA for Self-Eficacy Scales (n = 972)

	St. alpha	Alpha if item is dropped	Omega hierarchical	Omega Total	Mean (SD)	Item total correlation	Item total if item is dropped
SE for math	.8		.74	.83			
Item 1		.74			3.2 (.76)	.70	.61
Item 2		.71			2.9 (.82)	.76	.66
Item 3		.75			3.1 (.87)	.66	.59
Item 4		.77			2.9 (.95)	.61	.55
SE for writing	.75		.68	.8			
Item 5		.69			2.7 (.81)	.64	.55
Item 6		.70			3.0 (.80)	.62	.53
Item 7		.67			2.7 (.83)	.68	.59
Item 8		.71			2.9 (.83)	.60	.52
SE for reading	.79		.79	.81			
Item 9		.75			2.7 (.85)	.66	.58
Item 10		.72			2.9 (.80)	.73	.64
Item 11		.71			2.9 (.75)	.75	.66
Item 12		.78			2.5 (.80)	.57	.51
SE for studies natural	.82		.74	.86			
Item 13		.79			2.8 (.80)	.78	.66
Item 14		.77			2.8 (.81)	.79	.62
Item 15		.75			3.3 (.75)	.82	.66
Item 16		.76			3.4 (.71)	.74	.65

*Note: SE — self-efficacy*

#### Evidence Based on Relations to Other Variables

In this project, we did not measure self-efficacy using other self-efficacy scales. However, since each subscale is domain-specific but measures students’ self-efficacy, each subscale can be used as convergent evidence of validity. Discriminant evidence of validity was established using the SRL survey for elementary school students.

The correlations of the self-regulated learning strategies resulted in significant positive low correlations with the domain-specific self-efficacy scales, ranging from 0.21 to 0.37 (Table 3), which suggests that these scales measure different yet positively related constructs. The correlation estimates among domain-specific self-efficacy scales are moderate and significant. This suggests that they measure a similar trait, which contributes to the convergent evidence of validity. The correlations between self-efficacy in math and students’ math results (0.38) and self-efficacy in reading and students’ reading results (0.18) are positive and significant, which suggests that as students gain higher academic results, their self-efficacy increases^5^.

**Table 3:** Correlations Among Subscales of SRL Survey and Self-Efficacy Scales (n = 1,671)

	1	2	3	4	5	6	7
SRL	1						
SE math	.21***	1					
SE writing	.37***	.51***	1				
SE reading	.32***	.57***	.62***	1			
SE nature	.31***	.51***	.55***	.61***	1		
Math	–.04	.36***	.13***	.19***	.15***	1	
Reading	–.02	.26***	.17***	.18***	.17***	.46***	1
Mean (SD)	2.75 (0.59)	3.03 (0.66)	2.82 (0.62)	2.76 (0.63)	3.09 (0.60)	51.95 (9.82)	52.02 (9.01)

Note: ***p < .0001; **p < .001; *p < .05; SE — self-efficacy

#### Reliability

The reliability analysis was performed by estimating both Cronbach’s alpha and McDonald’s omega, which provide complementary and robust evidence of internal consistency (Deng & Chan, 2017). The reliability indices for each self-efficacy scale are good (Table 2), suggesting that the scales measure self-efficacy within their respective domains.

### Study 2: Self-Efficacy Scales for Middle School

As in Study 1, the internal structure of the surveys was examined using the EFA and CFA. The sample (*n =* 1,167) was randomly split into equal parts for EFA (*n* = 583) and CFA (*n* = 584). First, the EFA is described, followed by the CFA.

#### Exploratory and Confirmatory Factor Analyses

Before conducting the EFA, data correlations and assumptions of factorability and sphericity were checked. The inter-item correlations indicated small to medium positive correlations among items (.09–.78). The KMO factor adequacy overall estimate was 0.9, and the Bartlett test of sphericity was, χ^2^ (91) = 5810.75, *p* < .001, suggesting that it was appropriate to conduct a factor analysis.

The factor structure based on eigenvalues and the scree plots of the parallel analysis suggested a three-factor model, which corresponded with the three domains of foreign language, biology, and physics. The three-factor model indicated a good model fit, χ^2^ (52) = 165.73, *p* < .000, TLI = .96, RMSEA = .06, SRMR = .02.

The CFA was conducted on the second half of a sample (*n* = 584) to examine the three-factor structure of self-efficacy for foreign language, biology, and physics. The CFA indicated an excellent model fit, χ^2^ (74) = 126.56, *p* < .000, CFI = .99, TLI = .99, RMSEA = .03, SRMR = 0.03. The χ^2^/df coefficient resulted in an estimate of 1.71. In addition, all items had medium to large factor loadings. Table 4 reports on the item-level statistics and Appendix B includes the self-efficacy items for middle school.

**Table 4:** Reliability Indices and Item-Level Estimates after CFA (n = 584)

	St. alpha	Alpha if item is dropped	Omega hierarchical	Omega Total	Mean (SD)	Item total correlation	Item total if item is dropped
SE for foreign language	.93		.92	.94			
Item 1		.91			2.6 (.92)	.86	.83
Item 2		.91			2.4 (.96)	.88	.84
Item 3		.92			2.2 (.96)	.83	.80
Item 4		.92			2.6 (.91)	.82	.79
Item 5		.91			2.4 (.91)	.84	.81
SE for biology	.88		.85	.9			
Item 6		.85			2.5 (.80)	.81	.75
Item 7		.84			2.8 (.78)	.83	.77
Item 8		.85			2.7 (.76)	.79	.74
Item 9		.86			2.5 (.84)	.75	.70
SE for physics	.93		.9	.95			
Item 10		.92			2.5 (.87)	.83	.80
Item 11		.91			2.5 (.84)	.88	.84
Item 12		.91			2.5 (.83)	.87	.84
Item 13		.92			2.4 (.83)	.84	.81
Item 14		.92			2.4 (.89)	.80	.80

*Note: SE — self-efficacy*

#### Evidence Based on Relations with Other Variables

The initial convergent evidence was established by examining the correlations between domains of self-efficacy scales. Discriminant evidence of validity was examined using the metacognitive survey.

The correlations of all subscales with each other resulted in significant positive low estimates, ranging from .22 to .45 (Table 5), which provides convergent and discriminant evidence of validity. The correlation estimates among domain-specific self-efficacy scales are low, positive, and significant (.30–.45). The subscales of planning, monitoring, and reflection indicated positive, low yet significant correlations with self-efficacy for foreign language, biology, and physics (.21–.26). Low correlations contribute to the discriminant evidence of validity. Hence, each self-efficacy subscale resulted in positive, significant yet low correlations with the corresponding subject domains^6^. The results provide initial convergent and discriminant evidence of validity for self-efficacy scales in middle school.

**Table 5:** Correlations Among Subscales of Metacognition Survey and Self-Efficacy Subscales (n = 1,167)

	1	2	3	4	5	6	7	8	9
Planning	1								
Monitoring	.67***	1							
Reflection	.62***	.75***	1						
SE FL	.22***	.22***	.22***	1					
SE biology	.25***	.21***	.26***	.30***	1				
SE physics	.25***	.25***	.23***	.36***	.45***	1			
FL	.13***	.13***	.08**	.41***	.13***	.27***	1		
Biology	.17***	.16***	.13***	.21***	.34***	.28***	.53***	1	
Physics	.13***	.12***	.07**	.25***	.18***	.44***	.45***	.45***	1
Mean (SD)	2.75 (.63)	2.79 (.66)	2.68 (.74)	2.45 (.81)	2.62 (.66)	2.47 (.75)	4.01 (.77)	4.07 (.7)	3.71 (.86)

Note: ***p < .0001; SE --- self-efficacy; FL - foreign language

#### Reliability

Reliability was estimated using Cronbach’s alpha and McDonald’s omega. The reliability indices for each self-efficacy scale were high (α = .88–.93; ω_h_ = .85–.92; ω_t_ = .9–.95; Table 4), suggesting that the scales measure self-efficacy within their respective domains.

## Discussion

The goal of this study was to establish evidence of the construct validity of domain-specific self-efficacy scales developed for elementary and middle school students. The exploratory and confirmatory factor analyses results of self-efficacy scales for elementary school indicated that four out of six initial scales — self-efficacy in math, writing, reading, and natural studies — showed appropriate psychometric properties and a four-factor structure. The exploratory and confirmatory analyses of self-efficacy scales for middle school students also provided good evidence of construct validity by suggesting three distinct factors of self-efficacy for foreign language, biology, and physics. As a result, self-efficacy scales for elementary and middle school represent domain specificity, as suggested by Albert Bandura (2006).

Initial convergent evidence of validity was examined using correlations between self-efficacy domains in elementary and middle school samples. The results for the elementary school students indicated medium and significant correlations, suggesting that domain-specific self-efficacy scales are measuring a close construct. This was especially evident for self-efficacy in reading, which had medium correlations with all other domains ranging from .56 to .63. Conceptually, medium correlations between self-efficacy in reading and self-efficacy in other domains are understandable, because if students have good reading skills and high self-efficacy in reading, then they can read and, hopefully, understand what they are expected to do in other school subjects (Chen et al., 2021). In the middle school sample, the correlations between self-efficacy scales were significant yet low; the only correlation approaching medium estimates was between self-efficacy in biology and physics (.45), suggesting positive relationships between life and hard sciences. It can be concluded that convergent evidence of validity in this case is weak, because it should be established using another instrument measuring self-efficacy (AERA et al., 2014).

Correlations between self-efficacy scales and the SRL survey resulted in significant yet low correlations, which suggests that the two surveys measure related but distinct constructs for elementary school students. A similar pattern was observed for correlations between self-efficacy scales for middle school and subscales of the metacognitive survey: planning, monitoring, and reflection. These results contribute to the discriminant evidence of validity.

Finally, domain-specific self-efficacy scales were related to students’ academic performance in their respective domains, both in elementary and middle school samples. However, these relationships were low and the only correlation between self-efficacy in physics and students’ grades in physics approached a medium estimate (0.44). These results align with previous research studies reporting low to medium correlations between self-report measures of self-efficacy and academic achievement (DiBenedetto & Schunk, 2022).

Reliability analyses resulted in good estimates for each self-efficacy scale for elementary school as measured by Cronbach’s alphas and McDonald’s omegas. Similar analyses for middle school scales resulted in good estimates for self-efficacy for foreign language and biology, and excellent estimates for self-efficacy in physics. This evidence contributes to the internal consistency of each domain-specific self-efficacy scale for elementary and middle school.

## Conclusion

The evidence of construct validity suggests that the domain-specific self-efficacy scales for elementary and middle school students can be recommended for use both by researchers and practitioners. The scientific contribution of this paper is that it proposes domain-specific self-efficacy scales for elementary and middle school students, which have been developed using evidence-based guidelines in the fields of education and psychology, ensuring alignment with the federal state educational standards. As a result, the self-efficacy scales align with the requirements reflected in the legal documents for elementary and secondary education in Russia. Hence, these scales can facilitate future research studies in elementary and middle school settings to examine relationships between self-efficacy and academic achievement, as well as their contributions to student characteristics in line with studies in other countries (Grenner et al., 2021; Li et al., 2020).

In future research, scholars are invited to examine the role of self-efficacy in students’ academic results, as in a study examining its relation to computational thinking (Wei et al., 2021). Our initial examination of correlations between self-efficacy scales and students’ scores in subject domains resulted in positive yet low relations, which also requires further mediation and moderation analyses. We invite researchers to use sophisticated statistical techniques such as structural equation modeling (Kline, 2023) or cluster analyses (Wierzchon & Kłopotek, 2018) to examine relationships of self-efficacy with other variables and across multiple groups. In our own research, we found that self-efficacy in math and reading mediates the relationship between self-regulated learning and academic achievement and moderates the relationship between subjective well-being and academic results (Kanonire et al., 2023) in a sample of elementary students. Another area of future research could focus on establishing additional robust convergent and discriminant evidence of validity for self-efficacy scales and collecting evidence of response processes and consequences of testing as part of construct validity (AERA et al., 2014).

## Limitations

Even though the self-efficacy scales resulted in appropriate psychometric properties, this study has inherent limitations, which might affect the generalization of its results. To establish convergent evidence of validity, we relied on different domains of the same self-efficacy scales. While domain-specific self-efficacy scales for elementary school went through all phases of instrument development (Johnson & Morgan, 2016), scales for middle school were subject only to operationalization of self-efficacy (phase 1) and pilot testing (phase 2). It is recommended to collect additional data using self-efficacy scales for foreign language, biology, and physics to conduct model confirmation analyses (phase 3).

## Footnotes

1 Culture (O) is one of the constructs represented in the theoretical framework. It is a very complex construct that requires extensive research and the development of the measurement tools to capture its role in self- and socially regulated learning. Currently, there is some research evidence suggesting differences in self-efficacy beliefs between individualistic cultures (Western) and collectivist cultures (Eastern European, Asian). It seems that students from Eastern European and Asian countries have lower self-efficacy than students from Western countries (Klassen, 2004). Results for Russian children are inconclusive, because they showed higher self-efficacy than children from East Berlin (Klassen, 2004; Oettingen, 1995); however, verbal and social persuasion and high SES contributed to academic self-efficacy for Russian-speaking immigrant children in Germany, who come from former Soviet countries (Gebauer et al., 2021). These findings contribute to the need for cross-cultural self-efficacy studies between children from Russia and other countries.

2 Example item; *“I think about what I really need to learn before I begin a task.”*

3 Example item; *“I ask myself periodically if I am meeting my goals.”*

4 Example item; *“I ask myself if I learned as much as I could have once I finish a task.”*

5 Additional results on differences between boys and girls in different grades are provided in Appendix C.

6 Additional results on differences between boys and girls in different grades are provided in Appendix D.

## Appendix A

*Domain-Speci"c Self-Efficacy Scales for Elementary School*

**Instructions**: Подумай и ответь, можешь ли ты выполнить задания по разным предметам. Задания выполнять не нужно,

Поэтому отвечай честно, смог/смогла бы ты выполнить задания.

Шка ла отве тов : 1 — совсем не могу; 2 — могу НЕ очень хорошо; 3 — могу хорошо; 4 — могу очень хорошо.

**Table:**

Russian	English
**СЭ математика**	**Self-efficacy for mathematics**
Можешь ли ты решить пример по математике?	Can you solve a math equation?
Можешь ли ты решить задачу по математике?	Can you solve a math problem?
Можешь ли ты посчитать площадь прямоу-гольника?	Can you calculate the area of the rectangle?
Можешь ли ты назвать единицы длины?	Can you name the units of length?
**СЭ русский язык — письмо**	**Self-efficacy for writing**
Можешь ли ты что-то написать без ошибок под диктовку учителя?	Can you write without mistakes following the teacher’s dictation?
Можешь ли ты переписать без ошибок текст с доски или из книги в тетрадь?	Can you rewrite the text without mistakes from the board or textbook into your notebook?
Можешь ли ты написать изложение с состав-лением плана?	Can you write a composition following an outline?
Можешь ли ты написать короткое сочинение по картинке или на заданную тему?	Can you write a short essay describing a picture or topic?
**Литература — чтение**	**Self-efficacy for reading**
Можешь ли ты быстро (бегло) прочесть рас-сказ и понять при этом прочитанное?	Can you &uently read a story and understand its meaning?
Можешь ли пересказать главную мысль прочитанного рассказа?	Can you summarize the main idea of a story?
Можешь ли ты прочитать текст и ответить на вопросы учителя по этому тексту?	Can you read a story and answer the teacher’s questions about it?
Можешь ли ты понять значение новых слов в рассказе без помощи взрослого?	Can you understand new words in a story without help from adults?
**Окружающий мир**	**Self-efficacy for natural studies**
Можешь ли ты назвать основные группы жи-вотных и растений?	Can you name the main groups of animals and plants?
Можешь ли ты объяснить взаимосвязь между природой и человеком?	Can you explain the relationship between nature and humans?
Можешь ли ты рассказать о правилах гигиены?	Can you describe the rules of hygiene?
Можешь ли ты рассказать о правилах безо-пасности дома и на улице?	Can you describe the safety rules at home and outside?

## Appendix B

*Domain-Speci"c Self-e!cacy Scales for Middle School*

Instructions: Подумай и ответь, можешь ли ты выполнить задания по разным предметам. Задания выполнять не нужно,

Поэтому отвечай честно, смог/смогла бы ты выполнить задания.

Шка ла отве тов : 1 — совсем не могу; 2 — могу НЕ очень хорошо; 3 — могу хорошо; 4 — могу очень хорошо.

**Table:**

Russian	English
**СЭ иностранный язык**	**Self-efficacy for foreign language**
Можешь ли ты рассказать о себе или о собы-тии на ___ языке?	Can you talk about yourself or an event in the ____ language?
Можешь ли ты поддержать разговор на ___ языке с другими людьми (одноклассники, учителя, иностранцы)?	Can you speak with other people (peers, teachers, foreigners) in the ____ language?
Можешь ли ты написать сочинение или исто-рию на ___ языке?	Can you write an essay in the ____ language?
Можешь ли ты прочитать текст на ____ языке и понять его содержание без словаря?	Can you read a story in the ____ language and understand its meaning?
Можешь ли ты понять на слух разговор на ___ языке при просмотре видео?	Can you understand conversations when watching videos in the ____ language?
**СЭ биология**	**Self-efficacy for biology**
Можешь ли ты описать и классифицировать разные виды растений?	Can you describe and classify various types of plants?
Можешь ли ты описать и классифицировать разные виды животных?	Can you describe and classify various types of animals?
Можешь ли ты описать различные процессы, происходящие в организме человека?	Can you describe various processes happening in the human body?
Можешь ли ты применять научные методы наблюдения, измерения и эксперимента для описания живых существ?	Can you apply scientific methods of observation, measurement, and experimenting to describe living beings?
**СЭ физика**	**Self-efficacy for physics**
Можешь ли ты проводить прямые измерения физических величин (расстояние, время, объ-ем, температура и т.д.)?	Can you apply physical measurements such as distance, time, volume, temperature, etc.?
Можешь ли ты проводить исследование физи-ческих величин и делать выводы по результа-там исследования?	Can you conduct experiments using physical measurements and draw conclusions based on the results?
Можешь ли ты проводить опыты по наблюде-нию физических явлений или свойств тел?	Can you conduct experiments to observe physical phenomena or properties of bodies?
Можешь ли ты обосновывать выбор способа измерения или измерительного прибора при проведении исследований?	Can you justify the choice of measurement methods and instruments when conducting research?
Можешь ли ты распознавать проявление изученных физических явлений (кристаллизация, кипение, конденсация, взаимодействие магнитов и так далее) в окружающем мире?	Can you recognize physical phenomena such as crystallization, boiling, condensation, interaction of magnets, etc. in the world around you?

## Appendix C

**Table 1:** T-test Analysis of Fourth-Grade Students’ Academic Performance and Self-E"cacy by Gender (n = 1,671)

	Girls		Boys				
	*M*	*SD*	*M*	*SD*	*t(df)*	*p*	*Cohen’s D*
Math	51.41	9.95	52.51	9.66	–2.31 (1,668.3)	.02	–.11
Reading	52.39	9.11	51.65	8.89	1.68 (1,668.6)	.09	–
SE Math	2.95	.65	3.11	.66	–4.78 (1,666.8)	<.0001	–.23
SE Reading	2.75	.64	2.79	.62	–1.32 (1,668.5)	.18	–

*Note: SE — self-efficacy*

**Table 2:** T-test Analysis of Students’ Academic Performance and Self-E"cacy by Gender in Grades 4–6 (n = 1,167)

	Girls		Boys				
	*M*	*SD*	*M*	*SD*	*t(df)*	*p*	*Cohen’s D*
Math	3.96	.76	3.93	.78	.63 (1,389.2)	.53	–
Russian	3.99	.72	3.82	.71	4.62 (1,389.9)	<.0001	.25
Reading	4.35	.71	4.24	.71	2.93 (1,390)	.003	.16
SE Math	2.87	.68	3.03	.67	–4.49 (1,389.8)	<.0001	–.24
SE Russian	3.03	.59	2.81	.58	6.96 (1,389.9)	<.0001	.37
SE Reading	2.76	.64	2.79	.62	–1.12 (1,388.3)	.26	–

*Note: SE — self-efficacy*

**Table 3:** ANOVA Analysis of Students’ Academic Performance and Self-Efficacy by Grade (n = 1,167)

	4	5	6		ANOVA	
	*M*	*SD*	*M*	*SD*	*p*	*η^2^*
Math	4.16 (.74)	3.97 (.76)	3.76 (.77)	31.52	<.0001	.04
Russian	4.08 (.69)	3.96 (.72)	3.72 (.71)	30.85	<.0001	.04
Reading	4.46 (.65)	4.35 (.72)	4.10 (.71)	32.3	<.0001	.02
SE Math	3.17 (.65)	2.89 (.68)	2.82 (.66)	34.8	<.0001	.05
SE Russian	2.91 (.63)	2.92 (.59)	2.93 (.57)	.15	.86	–
SE Reading	2.91 (.65)	2.72 (.61)	2.74 (.62)	11.56	<.0001	.02

*Note: SE — self-efficacy*

## Appendix D

**Table 1:** T-test Analysis of Students’ Academic Performance and Self-E"cacy by Gender in Grades 7–9 (n = 1,392)

	Girls		Boys				
	*M*	*SD*	*M*	*SD*	*t(df)*	*p*	*Cohen’s D*
FL	4.16	.71	3.81	.79	7.96 (1,053.6)	<.0001	.47
Biology	4.19	.63	3.92	.76	6.5 (1,007.6)	<.0001	.39
Physics	3.81	.85	3.60	.87	4.18 (1,101.4)	<.0001	.25
SE FL	2.45	.81	2.44	.81	.30 (1,115.6)	.76	–
SE Biology	2.64	.64	2.59	.69	1.08 (1,075.8)	.28	–
SE Physics	2.39	.74	2.55	.77	–3.72 (1,095.9)	<.001	–.22

*Note: SE — self-efficacy; FL — foreign language*

**Table 2:** ANOVA Analysis of Students’ Academic Performance and Self-E"cacy by Grade (n = 1,392)

	*7*	*8*	*9*		ANOVA	
	*M (SD)*	*M (SD)*	*M (SD)*	*F (2, 1164)*	*p*	*η^2^*
FL	4.03 (.72)	3.96 (.77)	4.05 (.81)	1.58	.21	–
Biology	4.16 (.65)	4.04 (.73)	4.04 (.71)	3.57	.03	.006
Physics	3.76 (1.15)	3.68 (.71)	3.73 (.74)	0.86	.42	–
SE FL	2.44 (.83)	2.36 (.78)	2.6 (.84)	8.49	<.0001	.01
SE Biology	2.61 (.64)	2.66 (.64)	2.55 (.72)	2.57	.08	–
SE Physics	2.54 (.73)	2.37 (.73)	2.55 (.81)	8.21	<.001	.01

*Note: SE — self-efficacy; FL — foreign language*
